# Do histopathologic findings improve by increasing the sample size in cholecystectomies?

**DOI:** 10.1186/1477-7819-11-245

**Published:** 2013-10-01

**Authors:** Tumay Ozgur, Serhat Toprak, Ali Koyuncuer, Muhammed Guldur, Gurman Bayraktar, Mehmet Yaldiz

**Affiliations:** 1 School of Medicine, Pathology Department, Mustafa Kemal University, 31100 Serinyol-Hatay, Turkey; 2 Pathology Laboratory, Hatay Antakya State Hospital, Antakya-Hatay, Turkey

**Keywords:** Cholecystectomy, Sample size, Precancerous lesion

## Abstract

**Background:**

Gallbladder diseases present with cholelithiasis in a wide spectrum of adenomas and cancers. Two or three specimens are sampled in cholecystectomies in routine pathology practice. The aim of this study was to investigate the increase in frequency of histologic alterations in cholecystectomies, particularly precancerous lesions, by increasing the sample size to understand the carcinoma pathway.

**Methods:**

Cholecystectomies of 432 patients with pathology records and materials from two medical centers were collected, and two groups were created. Initial data with two or three samples were allocated to Group 1 and the new six samples with the initial ones were allocated to Group 2. Hematoxylin and eosin (H&E) sections were examined for histopathologic alterations, and periodic acid–Schiff (PAS) Alcian blue (pH 2.5) and high iron diamine (pH 2.5) stains were used to signify the mucin profile in case of metaplasias. For the comparison of findings, non-parametric tests, McNemar’s tests, chi-squared tests and Fisher’s exact test were performed.

**Results:**

Of the 432 patients, 308 (71.3%) patients were female and 124 (28.7%) patients were male. The mean age of patients was 47.9 ± 14.6 years. Cholesterolosis was observed in 95 (22%) patients in Group 1 and 108 (25%) patients in Group 2. Gallstones were detected in 255 (59%) of the cholecystectomies. There was a significant difference between Group 1 and Group 2 by increasing the sample size when we compared cholesterolosis, metaplasia and polyps (*P* <0.05). Cholecystitis and dysplasia rates were the same in both of the groups. There was no cancer determined.

**Conclusion:**

Increasing the sample size in cholecystectomies increased the diagnosis of some histologic alterations, but further studies with a larger number of samples over a longer period time might increase the ability to determine precancerous lesions and concomitants.

## Background

There are various lesions of the gallbladder that require surgical intervention. Gallstones and chronic cholecystitis are the most common pathologies, accompanied by cholesterolosis, adenomatous proliferation of mucous glands, metaplasia, dysplasia and hyperplasia [1]. Gallbladder cancer is the fifth most common malignant neoplasm of the gastrointestinal tract [2,3].

Metaplasia, dysplasia and hyperplasia are accepted as precursor lesions of the gallbladder; however, there is insufficient literature about the relationship of these lesions and gallbladder cancer. Detecting these lesions could provide a better understanding of carcinogenesis, risk factors and selecting prophylactic cholecystectomy patients. In this study, we investigated the possibility of determining increased rates of lesions, particularly precancerous lesions in gallbladders, which are the most common surgical material of daily pathology practice, with increased sample size.

## Methods

A prospective analysis was conducted at the Pathology Laboratory, Hatay Antakya State Hospital, and the Pathology Department, Mustafa Kemal University, Turkey, between October 2011 and May 2012, using the records and materials of laparoscopic cholecystectomies. The ethical committee on human research at Mustafa Kemal University approved the protocol for all human research.

The histopathologic examination consisted of an initial macroscopic examination of the specimen and suspicious areas with two or three samples. These specimens were allocated to Group 1.

The residual specimens were then collected in Mustafa Kemal University Pathology Department, and the fundus, body and neck of the gallbladders were sampled randomly. These new six samples with initial ones were allocated to Group 2.

These samples were then embedded in paraffin, sectioned, stained with hematoxylin and eosin (H&E), and examined under an Olympus BX53 (Olympus, Tokyo, Japan) light microscope. In case of existing metaplasia, the mucin profile was signified histochemically by neutral mucin, periodic acid–Schiff (PAS) Alcian blue (pH 2.5) and high iron diamine (pH 2.5). All histologic alterations described on the pathologists’ reports were included in the study. The presence of acute or chronic cholecystitis, gallstones, cholesterolosis, pyloric intestinal metaplasia, dysplasia, carcinoma, or polyps was analyzed as pathological data.

### Statistical analysis

Statistical evaluations were performed using SPSS 13.0 (IBM, Armonk, NY, USA) for Windows, and *P* <0.05 was considered statistically significant. For the comparison of findings, non-parametric tests, McNemar’s tests, chi-squared tests and Fisher’s exact test were performed.

## Results

Of the 432 cases, 308 (71.3%) patients were female and 124 (28.7%) patients were male. Patients’ ages varied from 18 to 84 years. The mean age of patients was 47.9 ± 14.6 years. Gallstones were obtained in 255 (59%) cholecystectomies. There were no stones in 177 (41%) gallbladders. The distributions of histologic alterations are shown in Table 1. Acute cholecystitis was observed in 31 (7.2%) patients, while chronic cholecystitis was observed in 401 (92.8%) patients in Group 1 and Group 2. There was no significant difference between them (*P* >0.05, kappa = 0.965).

**Table 1:** The distribution of histologic alterations within the groups

	**Group 1 (n/%)**	**Group 2 (n/%)**	***P***	**Kappa**
Acute cholecystitis	31 (7.2%)	31 (7.2%)	>0.05	0.965
Chronic cholecystitis	401 (92.8%)	401 (92.8%)		
Non-cholesterolosis	337 (78%)	324 (75%)	0.001	0.904
Cholesterolosis	95 (22%)	108 (25%)		
Non-metaplasia	407 (94.2%)	391 (91.2%)	0.0001	0.739
Pyloric metaplasia	23 (5.3%)	38 (8.8%)		
Intestinal metaplasia	2 (0.5%)	3 (0.7%)		
Non-polyp	422 (97.8%)	416 (96.6%)	0.031	0.76
Cholesterol polyp	7 (1.6%)	11 (2.5%)		
Fibroepithelial polyp	1 (0.2%)	2 (0.5%)		
Tubular adenoma	1 (0.2%)	1 (0.2%)		
Papillary adenoma	1 (0.2%)	1 (0.2%)		
Non-dysplasia	430 (99.5%)	430 (99.5%)	>0.05	1.0
Low grade dysplasia	2 (0.5%)	2 (0.5%)		
High grade dysplasia	0 (0%)	0 (0%)		
Carcinoma	0 (0%)	0 (0%)	>0.05	1.0

**Figure 1:**
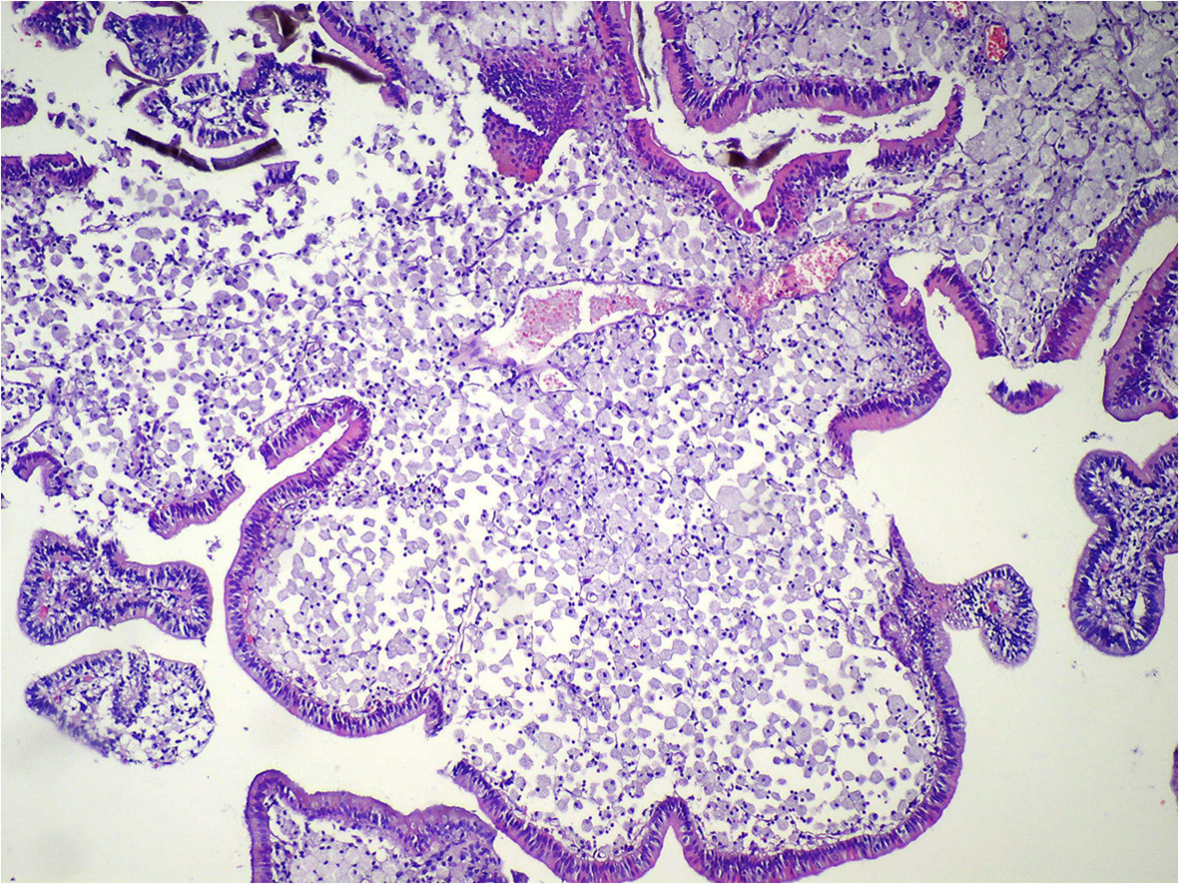
Cholesterol polyp (H&E ×100).

**Figure 2:**
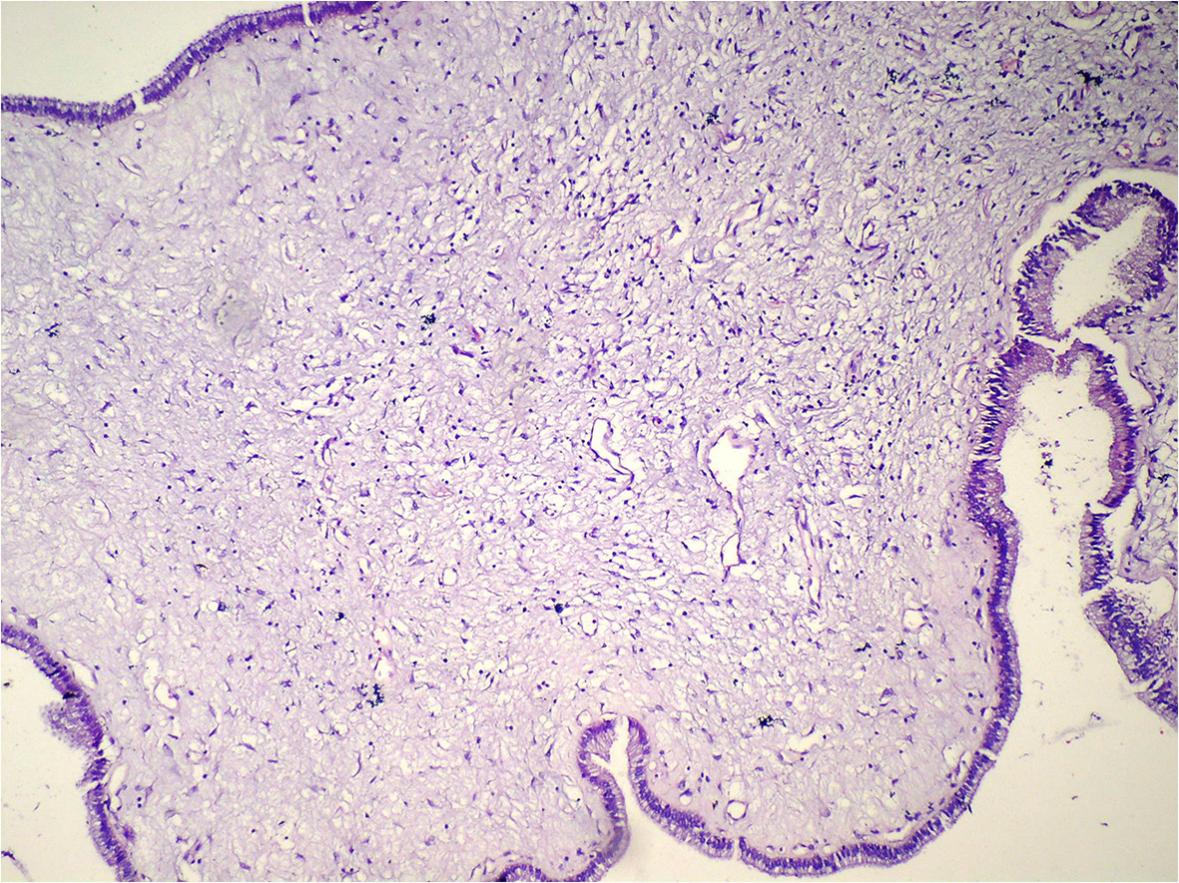
Fibroepithelial polyp (H&E ×100).

**Figure 3:**
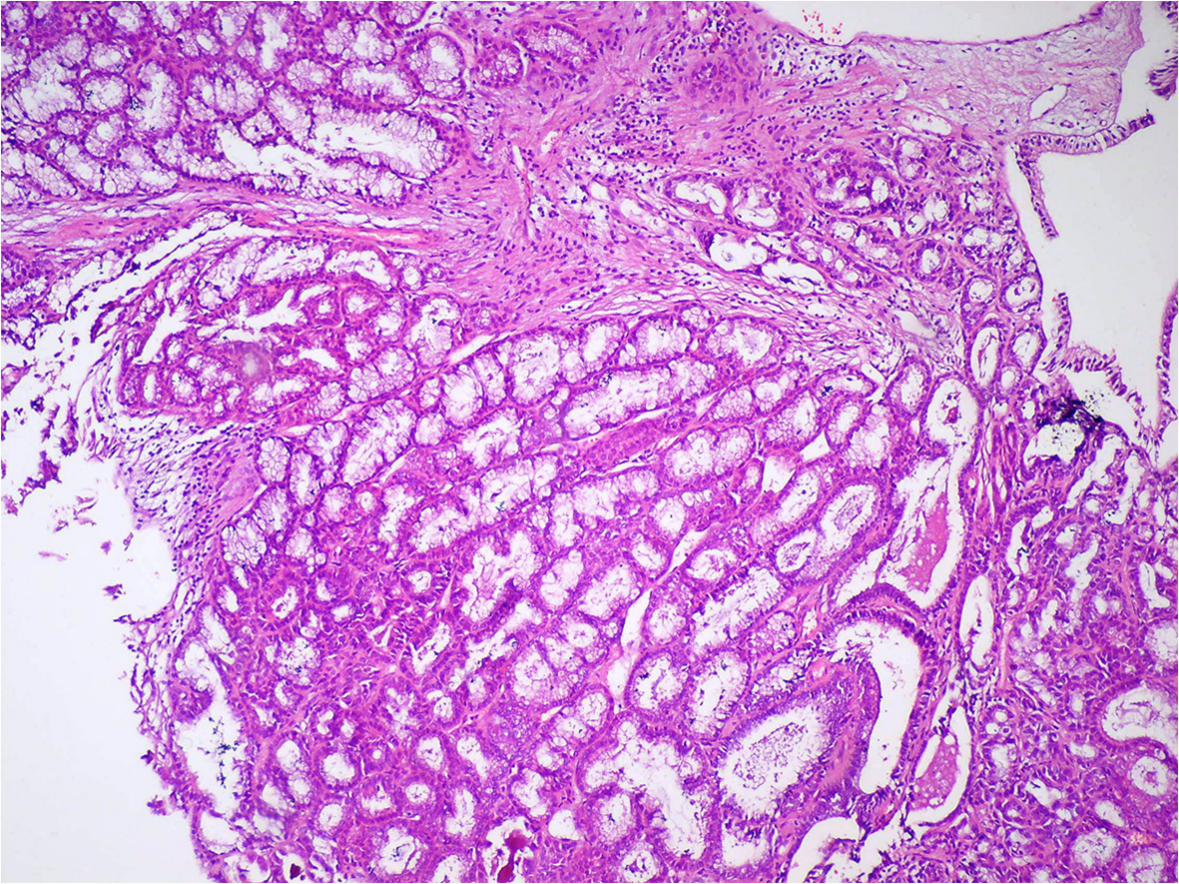
Tubular adenoma with low grade dysplasia (H&E ×100)

Cholesterolosis was observed in 95 (22%) patients in Group 1 and 108 (25%) patients in Group 2. There was a statistically significant difference between both of the groups (*P* = 0.001, kappa = 0.904). There were ten polyps (2.3%) in Group 1: seven (1.6%) were cholesterol polyps (Figure 1), one (0.2%) was a fibroepithelial polyp (Figure 2), one (0.2%) was a papillary adenoma, one (0.2%) was a tubular adenoma (Figure 3), and one (0.2%) case had both cholesterol and fibroepithelial polyps.

There were 16 (3.2%) polyps in Group 2: 11 (2.5%) were cholesterol polyps, two (0.5%) were fibroepithelial polyps, one (0.2%) was a papillary adenoma, one (0.2%) was a tubular adenoma, and one (0.2%) case had both cholesterol and fibroepithelial polyps. There was a statistically significant difference between the two groups (*P* = 0.031, kappa = 0.762).

**Figure 4:**
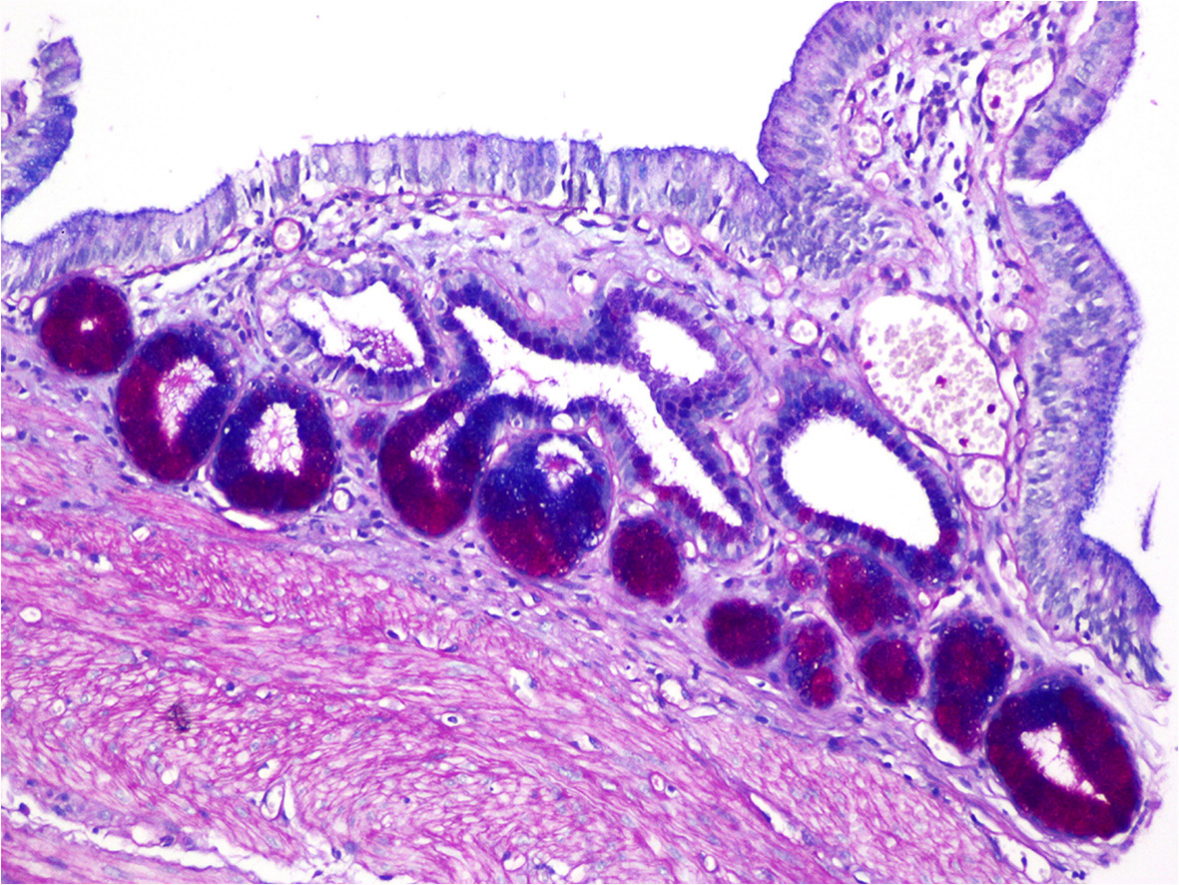
Pyloric metaplasia in mucosa (PAS Alcian blue ×100).

Metaplasia was observed in 25 (5.8%) of the cases in Group 1: 23 (5.3%) of them were pyloric metaplasia (Figure 4), while two (0.5%) were intestinal. Forty-one (9.5%) cases in Group 2 had metaplasia: 38 (8.8%) were pyloric and three (0.7%) were intestinal. There was a significant difference between the two groups when comparing metaplasia (*P* = 0.0001, kappa = 0.739).

We did not diagnose any carcinoma in this study period. However, there was mild dysplasia in two (0.5%) of the cases in both of the groups, and there was no significant difference between them (*P* >0.05, kappa = 1.0).

## Discussion

Gallbladder carcinoma is rarely diagnosed in precancerous stages due to its occult evolution and non-specific symptoms. There is little knowledge about its etiology or pathogenesis, and determining precursor lesions is difficult. Environmental and genetic risk factors are important in carcinoma development. There are two genetic alterations: adenoma-carcinoma sequence and the metaplasia-dysplasia-carcinoma sequence [4-6]. Gallbladder carcinoma is incidentally discovered during cholecystectomies performed for cholelithiasis or cholecystitis. Diagnosis and sampling of small-sized and flat epithelial lesions in early stages is difficult; therefore, histopathologic evaluation of cholecystectomy specimens is important [7]. Pyloric metaplasia is more frequent than intestinal metaplasia in cholecystectomies [4,5,8]. We observed pyloric metaplasia predominance in both of our study groups with 23 (5.3%) cases in Group 1 and 38 (8.8%) cases in Group 2, similar to the literature. Metaplastic epithelium is more susceptible to malignant transformation than the normal epithelium [9]. We determined low grade dysplasia in two (0.5%) cases, and there was no increase in the prevalence of this lesion with the increased sample size. Besides this observation, there was no significant relationship between metaplasia and dysplasia. In contrast to this study, Mazlum *et al*. observed metaplasia in 18 out of 24 dysplastic gallbladders either adjacent to or within the dysplastic epithelium [10]. Meirelles-Costa *et al*. also found an association between pyloric metaplasia and dysplasia, supporting the metaplasia-dysplasia-carcinoma sequence [11].

The discrepancies of our results might be related to the smaller population of our study group and the short interval of the study. On the other hand, this disease progresses slowly and a longer follow-up period should be planned to obtain results that are similar to the current literature.

Gallbladder polyps present with conditions differing from cholesterol polyps to adenomas. Cholesterol polyps are one of the most frequent types stated in various studies [10,12,13]. Similar to the literature, we detected 16 (3.2%) polyps in Group 2 with the increased sample size, and 11 (2.5%) of those were cholesterol polyps. This variant of polyps is defined as the polypoid form of cholesterolosis [14]. Cholesterolosis is characterized by the accumulation of cholesterol esters in epithelioid histiocytes in the hyperplastic mucosa of the gallbladder [15]. We found cholesterolosis in 95 (22%) patients in Group 1 and 108 (25%) patients in Group 2, with a significant difference between them. These ratios were higher when compared to the Mazlum *et al*. study, which reported 13.3% of cholesterolosis cases from the Western part of Turkey [10]. Parallel to our study, Bolat *et al*. detected cholesterolosis in 14 (18.7%) patients in their study [16]. In our geographical area, the population has a traditional Eastern Mediterranean diet, which is high in carbohydrates and fat, and could result in metabolic syndrome, particularly in women with a higher body mass index (BMI). We also found more cholesterol polyps and cholecystitis in females compared to males.

Adenomas are rare lesions of the gallbladder, and can be identified as tubular, papillary and tubulopapillary [17]. We observed two (0.4%) adenomas in our study: one (0.2%) case was tubular and the other (0.2%) papillary. There was low grade dysplasia within the tubular adenoma, which is described frequently in other studies [9,17]. However, we did not detect any carcinoma focus in our cases. This could be related to the rare malignant transformation and size of the adenoma in gallbladders when compared to other gastrointestinal organs [18]. On the other hand, we should keep in mind that the development of laparoscopic surgical techniques and the recommendation of cholecystectomy for cholelithiasis and cholecystitis with long evolution have reduced the cancer cases [19].

We determined higher ratios of polyp, cholesterolosis and metaplasia when the sample size was increased in Group 2. The difference of the results of the two groups in non-neoplastic lesions might be due to the neglect of the sampling and evaluation of the lesions. However, preneoplastic lesions, such as metaplasia and dysplasia, cannot be determined with macroscopic examination, and at this point increasing the number of specimens becomes essential. Parallel to this study, the importance and value of macroscopic sampling has been highlighted in other studies [4,5]. Mukhopadhyay *et al*. also determined that in most cases of dysplasia with a coexistent metaplastic lesion, dysplasia arises from precursor lesions, not directly from an inflammatory background [4]. Therefore, sampling of the epithelium, unrelated with injured and inflamed zones in gallbladders, and with no mucosal irregularity or thickness for noticing dysplasia, comes into question. On the other hand, the detection of focal epithelial changes is a result of the number of sections examined. Duarte *et al*. included fewer patients but used far more extensive sampling. They found that a single random histologic section detected less than one third of metaplasias and dysplasias in the gallbladder, and used whole mapping of the gallbladders in their study [5].

## Conclusions

All histologic findings, with the exception of cholelithiasis and cholecystitis, had low incidence upon examination of the cases. However, there was a significant difference among metaplasia, polyp and cholesterolosis evaluation between the two groups, suggesting that the increased sample size would raise the possibility of determining more histologic lesions. Further studies with a greater number of samples and a longer follow-up period might increase the detection of precancerous lesions and concomitants.

## Abbreviations

BMI: Body mass index; H&E: Hematoxylin and eosin; PAS: Periodic acid–Schiff.

## Competing interests

The authors declare that they have no competing interests.

## Authors’ contributions

TO designed the study, searched the literature and drafted the manuscript. ST sampled the materials and prepared the sections. AK searched the literature, examined the materials and prepared the figures. MG and GB examined the materials. MY participated in study design and coordination. All authors read and approved the final manuscript.

## References

[B1] Rosai J Rosai J From Gall bladder and extrahepatic bile ducts Rosai and Ackerman’s Surgical Pathology 2000 9 New York, NY: Mosby 1035 1060 Volume 1.

[B2] Bartlett DL Gall bladder cancer Semin Surg Oncol 2000 19 145 155 10.1002/1098-2388(200009)19:2<145::AID-SSU7>3.0.CO;2-6 11126379

[B3] Lowenfels AB Maisonneuve P Boyle P Zatonski WA Epidemiology of the gall bladder cancer Hepatogastroenterology 1999 46 Suppl 27 1529 1532 10430289

[B4] Mukhopadhyay S Landas SK Putative precursors of gall bladder dysplasia: a review of 400 routinely resected specimens Arch Pathol Lab Med 2005 129 386 390 15737036 10.5858/2005-129-386-PPOGDA

[B5] Duarte I Llanos O Domke H Harz C Valdivieso V Metaplasia and precursor lesions of gall bladder carcinoma. Frequency, distribution and probability of detection in routine histologic samples Cancer 1993 72 1878 1884 10.1002/1097-0142(19930915)72:6<1878::AID-CNCR2820720615>3.0.CO;2-2 8364865

[B6] Albores-Saavedra J Alcantra-Vazquez A Cruz-Ortiz H Herrera-Goepfert R The precursor lesions of invasive gall bladder carcinoma. Hyperplasia, atypical hyperplasia and carcinoma in situ Cancer 1980 45 919 927 10.1002/1097-0142(19800301)45:5<919::AID-CNCR2820450514>3.0.CO;2-4 7260842

[B7] Sasatomi E Tokunaga O Miyazaki K Precancerous conditions of gall bladder carcinoma: overview of histopathologic characteristics and molecular genetic findings J Hepatobiliary Pancreat Surg 2000 7 556 567 10.1007/s005340070004 11180887

[B8] Stancu M Caruntu ID Gıuşca S Dobrescu G Hyperplasia, metaplasia, dysplasia and neoplasia lesions in chronic cholecytitis- a morphologic study Rom J Morph Embryol 2007 48 Suppl 4 335 342 18060182

[B9] Mukada T Andoh N Matsushiro T Precancerous lesions of the gallbladder mucosa Tohoku J Exp Med 1985 145 387 394 10.1620/tjem.145.387 4024073

[B10] Mazlum M Dilek FH Yener AN Tokyol C Aktepe F Dilek ON Profile of gallbladder diseases diagnosed at Afyon Kocatepe University: a retrospective study Turk Patoloji Derg 2011 27 Suppl 1 23 30 21469423

[B11] Meirelles-Costa AL Bresciani CJC Perez RO Bresciani BH Siqueira SA Cecconello I Are histologic alterations observed in the gall bladder precancerous lesions? Clinics 2010 65 Suppl 2 43 50 10.1590/S1807-59322010000200005 PMC2827700 20186297

[B12] Terzi C Sökmen S Seçkin S Albayrak L Uğurlu M Polypoid lesions of the gall bladder: report of 100 cases with special reference to operative indications Surgery 2000 127 622 627 10.1067/msy.2000.105870 10840356

[B13] Escalona A León F Bellolio F Pimentel F Guajardo M Gennero R Cruz JP Viviani P Ibáñez L Gallbladder polyps: correlation between ultrasonographic and histopathologic findings Rev Med Chil 2006 134 1237 1242 17186092 10.4067/s0034-98872006001000004

[B14] Jørgensen T Jensen KH Polyps in the gallbladder. A prevalence study Scand J Gastroenterol 1990 25 281 286 2320947

[B15] Owen CC Bilhartz LE Gallbladder poyps, cholesterolosis, adenomyomatosis and acute acalculouscholcytitis Semin Gastrointest Dis 2003 14 178 188 14719768

[B16] Bolat F Kayaselcuk F Nursal TZ Bal N Tuncer I The correlation of the histopathologic findings by increasing the sample size in cholecystectomies Turk J Pathol 2007 23 Suppl 3 137 142

[B17] Hansel DE Maitra A Argani P Pathology of the gall bladder: a review Cur Diagn Pathol 2004 10 304 317 10.1016/j.cdip.2004.03.006

[B18] Roa I de Aretxabala X Araya JC Roa J Preneoplastic lesions in gallbladder cancer J Surg Oncol 2006 93 615 623 10.1002/jso.20527 16724345

[B19] Orth K Beger HG Gall bladder carcinoma and surgical treatment Langenbecks Arch Surg 2000 385 8 501 508 10.1007/s004230000178 11201005

